# PET-CT imaging of pulmonary inflammation using [^68^Ga]Ga-DOTA-TATE

**DOI:** 10.1186/s13550-022-00892-0

**Published:** 2022-04-08

**Authors:** Emmi Puuvuori, Francesco Liggieri, Irina Velikyan, Elena Chiodaroli, Jonathan Sigfridsson, Hampus Romelin, Sofie Ingvast, Olle Korsgren, Gry Hulsart-Billström, Gaetano Perchiazzi, Olof Eriksson

**Affiliations:** 1 Science for Life Laboratory, Department of Medicinal Chemistry, Uppsala University, Dag Hammarskjölds väg 14C, 3tr, 751 83 Uppsala, Sweden; 2 Hedenstierna Laboratory, Department of Surgical Sciences, Uppsala University, Uppsala, Sweden; 3 Department of Immunology, Genetics and Pathology, Uppsala University, Uppsala, Sweden

**Keywords:** [^68^Ga]Ga-DOTA-TATE, Inflammation imaging, PET, Macrophage, Lung inflammation

## Abstract

**Purpose:**

In the characterization of severe lung diseases, early detection of specific inflammatory cells could help to monitor patients’ response to therapy and increase chances of survival. Macrophages contribute to regulating the resolution and termination of inflammation and have increasingly been of interest for targeted therapies. [^68^Ga]Ga-DOTA-TATE is an established clinical radiopharmaceutical targeting somatostatin receptor subtype 2 (SSTR 2). Since activated macrophages (M1) overexpress SSTR 2, the aim of this study was to investigate the applicability of [^68^Ga]Ga-DOTA-TATE for positron emission tomography (PET) imaging of M1 macrophages in pulmonary inflammation.

**Methods:**

Inflammation in the pig lungs was induced by warm saline lavage followed by injurious ventilation in farm pigs (*n* = 7). Healthy pigs (*n* = 3) were used as control. A 60-min dynamic PET scan over the lungs was performed after [^68^Ga]Ga-DOTA-TATE injection and [^18^F]FDG scan was executed afterward for comparison. The uptake of both tracers was assessed as mean standardized uptake values (SUV_mean_) 30–60-min post-injection. The PET scans were followed by computed tomography (CT) scans, and the Hounsfield units (HU) were quantified of the coronal segments. Basal and apical segments of the lungs were harvested for histology staining. A rat lung inflammation model was also studied for tracer specificity using lipopolysaccharides (LPS) by oropharyngeal aspiration. Organ biodistribution, ex vivo autoradiography (ARG) and histology samples were conducted on LPS treated, octreotide induced blocking and control healthy rats.

**Results:**

The accumulation of [^68^Ga]Ga-DOTA-TATE on pig lavage model was prominent in the more severely injured dorsal segments of the lungs (SUVmean = 0.91 ± 0.56), compared with control animals (SUVmean = 0.27 ± 0.16, *p* < 0.05). The tracer uptake corresponded to the damaged areas assessed by CT and histology and were in line with HU quantification. The [^68^Ga]Ga-DOTA-TATE uptake in LPS treated rat lungs could be blocked and was significantly higher compared with control group.

**Conclusion:**

The feasibility of the noninvasive assessment of tissue macrophages using [^68^Ga]Ga-DOTA-TATE/PET was demonstrated in both porcine and rat lung inflammation models. [^68^Ga]Ga-DOTA-TATE has a great potential to be used to study the role and presence of macrophages in humans in fight against severe lung diseases.

**Supplementary Information:**

The online version contains supplementary material available at 10.1186/s13550-022-00892-0.

## Introduction

The cascade of the immune reaction in various lung diseases is a fascinatingly coordinated procedure in order for the tissue to return to homeostasis. However, to monitor pathogenic processes and characterize new lung diseases more information about specific inflammatory cell activation is required. Most of the clinically available methods are still limited to blood sampling, even though the immune cells in the blood circulation do not automatically correspond with the amount or phenotypes of the tissue resident cells [1]. Considering lung diseases, invasive biopsies and histopathology are difficult, risky, and time-consuming approaches, which are also prone to sampling errors. One of the safest options to assess local inflammatory process is noninvasive imaging with positron emission tomography in combination with computed tomography (PET-CT). The majority of the clinical inflammation and infection imaging is heavily reliant on [^18^F]FDG/PET-CT imaging. It is a great tool to serve as increased metabolism marker, but does not offer any information about specific cell activation and can be prone to false positive findings [2]. The use of [^18^F]FDG for lung inflammation imaging is also limited by the high uptake in the myocardium, which can complicate the interpretation of the images and full understanding of the severity of the disease [3].

Another clinically available PET radiotracer [^68^Ga]Ga-DOTA-TATE is commonly used for somatostatin receptor (SSR) positive neuroendocrine tumor imaging. Somatostatin is an inhibitory peptide hormone that is produced in multiple locations in the body. Its main function is to regulate the endocrine system and modulate neurotransmission in central nervous system (CNS) [4]. Expectedly, in [^68^Ga]Ga-DOTA-TATE imaging of healthy humans, the highest SSR mediated uptake is seen in endocrine organs spleen, pancreas, adrenal-, thyroid- and pituitary gland. Non-specific uptake is increased in the liver and kidneys, since the peptide is small enough to be both filtered and reabsorbed by the nephrons. Moderate uptake has also been found in bone in degenerative diseases and growth plates of children since osteoblasts express somatostatin receptor subtype 2 (SSTR 2) [5]. No uptake in the lungs of healthy humans has been detected, making [^68^Ga]Ga-DOTA-TATE an excellent candidate to image lung inflammation [4].

Five subtypes of SSRs (SSTR 1- SSTR 5) have been discovered with a wide expression pattern in both normal tissues and tumors [6]. Since the naturally occurring somatostatin has low stability, more stable synthetic analogues have been developed with varied binding affinity to the different subtypes. [^68^Ga]Ga-DOTA-TATE has the highest affinity (IC_50_ 0.2 nM) [7] for SSTR 2, which is the most common subtype found on most neuroendocrine tumors [4]. However, pro-inflammatory M1 macrophages also highly express SSTR 2 [8], and several recent clinical studies have successfully used [^68^Ga]Ga-DOTA-TATE conducted to visualize inflammatory foci in atherosclerosis [8–10].

Macrophages are versatile immune cells that play a fundamental role in inflammation. In lung tissue, the macrophages reside in the lung interstitium and alveoli and through their functions in tissue repairing and modeling they contribute to restoring gas exchange back to normal [11]. During both sterile inflammation and pathogen triggered infection, tissue macrophages recruit cells from both the blood stream, hematopoietic organs, and through local proliferation. Macrophages are typically divided into three phenotypes, M0 (undifferentiated) as the Naïve state, M1 as the pro-inflammatory (or classical) and M2 as anti-inflammatory (or alternative) type [12]. The activation of M0 to M1 macrophages can be promoted by lipopolysaccharides (LPS), interferon gamma (IFN-), and granulocyte–macrophage colony-stimulating factor (GM-CSF) and after differentiation, the M1 cells secrete pro-inflammatory cytokines, e.g., interleukins (IL-1, IL-12, IL-18 and IL-23) and tumor necrosis factor alpha (TNF-α) [13]. Although the functions of macrophages are designed to protect us, the host, prolonged activation may generate tissue destruction and endanger cardiovascular health [14–16].

Thus, the literature indicates that [^68^Ga]Ga-DOTA-TATE can be used for PET imaging of M1 polarized macrophages, via binding to the SSTR2 on their surface. However, this mode of action has not been yet been demonstrated in vivo, due to the obvious difficulties in performing blocking studies or correlative staining in biopsies from human subjects.

The purpose of this study was to further evaluate the performance of [^68^Ga]Ga-DOTA-TATE as an macrophage marker, by in vivo PET imaging in established animal models of pulmonary inflammation, in comparison with invasive assessment parameters such as lung function, correlative staining and in vivo blocking.

## Materials and methods

### Radiosynthesis of [^68^Ga]Ga-DOTA-TATE

The ^68^Ga-labeling was conducted using fractionation method [17]. The detailed procedures are shown in Additional file 1. The radiochemical purity was 98.9 ± 0.5%.

### Pilot study in LPS lung inflammation in rats

A pilot experiment was performed with rats (Sprague Dawley, *n* = 3, weight: 270–293 g) induced with lung inflammation and injury by treatment with lipopolysaccharides (LPS, from salmonella typhosa, 25 mg Sigma-Aldrich) (oropharyngeal aspiration, 9.5 mg/mL in PBS, 70 µL/rat). The rats were scanned by magnetic resonance imaging (MRI) and CT (see protocols below) at baseline, 24-h, 48-h and 72-h post-administration of LPS to follow the development of inflammation and assist determination of the most optimal time point for [^68^Ga]Ga-DOTA-TATE injection and PET imaging.

Afterward, another set of rats (*n* = 3, weight: 273–300 g) was administered approximately 15 MBq [^68^Ga]Ga-DOTA-TATE intravenously 24 h after LPS treatment under 3% isoflurane anesthesia. The rats were kept under gas anesthesia for 60 min and then were euthanized by CO_2_. Lung tissue was collected postmortem and divided into two parts—one embedded in OCT and used for ex vivo autoradiography to study [^68^Ga]Ga-DOTA-TATE binding, and the other part fixed in 4% paraformaldehyde (PFA) for correlative histology (see below for details).

### [^68^Ga]Ga-DOTA-TATE PET-MRI + CT imaging of rats with lung inflammation pilot study

The distribution of [^68^Ga]Ga-DOTA-TATE was visualized in Sprague Dawley rats (*n* = 3) with LPS induced lung inflammation, on PET-MRI and CT. The rats were given LPS administration as described in the protocol above. For the imaging, they were initially anesthetized with isoflurane 5%, which was maintained at 3% throughout the scans. The rats were placed on the camera bed with heated pad and injected with [^68^Ga]Ga-DOTA-TATE (15 MBq), and a 30-min dynamic PET scan (nanoPET-MRI, Mediso Medical Imaging Systems, Hungary) over the lungs (283 × 283 × 321 matrix) was acquired. The MRI was executed using gradient echo 2D axial sequence (TR/TE 260/4.1 ms, 0.4-mm spatial resolution, 19 slices) and CT scan (50 kVp, 610 µA) of 7 min was acquired. The rats were euthanized by CO_2_ after the scans and the PET images were reconstructed for attenuation, dead time, positron range and scatter correction (Tera-Tomo™ 3D: 0.3-mm^3^ spatial resolution, OSEM, Monte Carlo DOI estimation, 4 iterations, 6 subsets, voxel size 0.4 mm^3^).

### Lung inflammation model and ex vivo biodistribution of [^68^Ga]Ga-DOTA-TATE in rats

For the full study, rats (*n* = 15, weight: 236–290 g) were divided into three groups: LPS, blocking and control with 5 animals/group. LPS was given as oropharyngeal aspiration (9.5 mg/mL in PBS, 70 µL/rat as above) on the LPS baseline and LPS blocking groups 24 h before ex vivo biodistribution. Based on the pilot study described above, LPS induced lung inflammation was apparent already after 24 h, and this time-point was thus selected as endpoint for the full study. In the blocking group, 1.5 mg/kg of somatostatin analogue octreotide (Sandostatin, 500 μg/mL, Novartis) was injected 30 min prior to [^68^Ga]Ga-DOTA-TATE injection. The rats were anesthetized with isoflurane (4%) and [^68^Ga]Ga-DOTA-TATE was administered by a bolus injection (8–27 MBq) via the tail vein. The rats were sacrificed by CO_2_ 1 h after injection and lungs, thymus, muscle, myocardium, blood, trachea, esophagus and stomach were excised and measured on gamma counter (1480 Wizard, Wallac Oy, Turku, Finland). The data was decay- and weight-corrected to SUV and expressed as a ratio to heart muscle.

Immediately after the gamma counter measurements, parts of the lungs, thymus and muscle were embedded in OCT media and snap frozen. The remaining parts of lungs and thymus were embedded into cassettes (Q Path MicroStar 2, Avantor) and fixed in PFA for histology for 24 h and later transferred to 70% EtOH at 4 °C.

## Ex vivo autoradiography of rat tissues

Coronal slices of the organs (lungs, thymus and muscle) were cut to 20-μm-thick sections using a cryostat (Cryostar NX70, Thermo Fisher Scientific). The sections were mounted on Superfrost Plus microscope slides (Menzel-Gläser, Germany) and exposed to a recently erased storage phosphor screen (BAS-MS, Fujifilm) for two half-lives of ^68^Ga. Subsequently, the imaging plates were scanned on a phosphor imager (Amersham Typhoon FLA 9500 Phospor Imager, GE) with 4000 sensitivity and 25-µm pixel size. A set of three calibration standards were created for quantification by pipetting 5-μL drops of the original stock solution onto absorbent chromatography paper. The images were analyzed in ImageJ (National Institutes of Health, US) software.

## Histology of rat tissues

All rat biopsies were fixed in PFA for 24 h, then washed and switched into 70% EtOH at 4 °C until further processing. The biopsies were embedded in paraffin, sectioned into 4-µm sections and stained for hematoxylin/eosin (H/E), Masson’s trichrome (MTC), Sirius Red (SIR) according to standard methods, as well as immunohistological staining (IHC) for macrophage marker CD68 (primary antibody KP1, DAKO, Glostrup, Denmark). Antigen retrieval, IHC staining and development by the Dako EnVision (DAB) system were performed according to the manufacturer’s instructions using antigen retrieval at pH 9, 1:100 primary antibody dilution and 30 min incubation time.

### Lung inflammation induction in pig

Pigs (*n* = 10, weight: 25–30 kg, Swedish landrace) were transported to Uppsala University on the day of the study. Anesthesia was induced by intramuscular administration of tiletamine–zolazepam. Then, the pig was intubated for controlled ventilation, while anesthesia was continuously supported with intravenous ketamine, fentanyl and midazolam as described previously in detail [18].

During instrumentation and the following phases of the experiment, the animal was ventilated in volume control mode, with an initial tidal volume (Vt) of 6–8 mL/kg and a Respiratory Rate (RR) = 20/min in order to maintain normocapnia [38–42 mmHg] in arterial blood, Positive End-Expiratory Pressure (PEEP) = 5 [cmH_2_O], FiO_2_ = 0.5. This baseline pattern of ventilation was modified during the sequence to induce lung injury (as described below) and re-started after the induction of damage.

Of the ten pigs used in this study, seven were induced with lung inflammation while three were untreated. For this study, an established dual-hit model of lung injury was used, consisting in repeated lung lavages (for removing lung surfactant) followed by the application of an injurious pattern of mechanical ventilation in order to induce the so-called ventilator induced of lung injury (VILI) [19] using a previously described experimental procedure [18].

Briefly, repeated lavages were performed with 35 mL/kg warm NaCl 0.9%. The lung function was assessed after each three lavages by monitoring ventilator readout and arterial blood gases (p/f ratio, Vd/Vt ratio, oxygen saturation). Maximum eight lavages were performed, or until the p/f ratio was recorded as below 100 [mmHg] (severe ARDS). Then, injurious ventilation was started by applying a pressure control mode with the following parameters: PEEP = 0 [cmH_2_O]; airway Peak pressure = 35 [cmH_2_O]; RR = 30/min; FiO_2_ = 1. In order to induce VILI, this pattern was continued for 1 h; during injurious ventilation the animal reached a tidal volume > 12 mL/kg body weight. In the phases of the experiment following the induction of lung injury, while the pattern of breathing was the same used at baseline, the FiO_2_ could be kept at 1 in order to cope with the alteration of gas exchange determined by the experimental ARDS and aiming to provide a sufficient oxygen delivery to peripheral tissues.

### [^68^Ga]Ga-DOTA-TATE PET-CT imaging of pigs with lung inflammation

First, the pigs were placed on the scanner bed on supine position. An attenuation CT scan (100 kV, 80–400 mA, noise index 10, rotation 0.5’’, full spiral, slice thickness 3.75 mm, pitch 0.98:1, recon diameter 50 mm) was acquired using a digital 4-ring system, 64-slice CT with a 198-mm axial field of view (FOV). A dynamic PET (Discovery MI, GE Healthcare) scan of 60 min (30 frames: 12 × 10 s’’, 6 × 30 s’’, 5 × 2 min’, 5 × 5 min’, 2 × 10 min’) was performed over the lungs after injection of [^68^Ga]Ga-DOTA-TATE (2 MBq/kg) *n* = 7 lavage, 3 control. Around 3 h after the [^68^Ga]Ga-DOTA-TATE scan was finished (to allow for 68Ga decay), the pig was injected with [^18^F]FDG (*n* = 3 lavage, 1 control) and another dynamic scan of 60 min was obtained. During the dynamic scans arterial blood samples were collected and the activity was measured on a well counter to obtain the tracer volume in plasma and whole blood. The lung perfusion was visualized by late arterial (17 s) and venous phase contrast-enhanced CT (70 mL of Omnipaque 350 + 40 mL NaCl, 3.5 mL/s, bolus tracking on descending aorta 100 HU threshold). The PET images were reconstructed with an iterative VPFX-S algorithm (GE Healthcare) (3 iterations, 16 subsets, 3-mm postfilter and 256 × 256 matrix).

After the PET scans, the animal was euthanized by intravenous KCl under deep anesthesia. Biopsies were taken from the lung (right and left, apical and basal parts), spleen and liver, both for snap freezing and fixation in formalin. The formalin biopsies were embedded, sectioned, and stained by several routine methods, e.g., H/E.

### PET-CT pig image data analysis

**Fig. 1:**
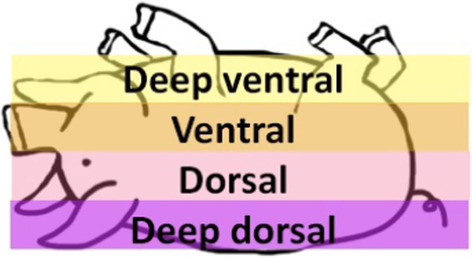
Illustration of the segmentation of the pig PET-CT VOIs. Pig laying on supine position

The delineation of volumes of interest (VOIs) were manually drawn over the lungs on standard uptake value (SUV) corrected transaxial projections using PMOD software (PMOD Technologies LLC, Zürich, Switzerland). The segmentations were divided according to the anatomical location: from deep ventral segment being the furthest ventral segment and deep dorsal segment being the furthest dorsal segment of the animal (Fig. 1). Hounsfield unit (HU) values were obtained from CT images. All the diagrams were summarized on GraphPad Prism (GraphPad Software Inc., La Jolla, CA, USA), and all the data are presented as the mean and standard deviation of the mean. Comparisons between different segments were assessed by Student's t test (two-tailed), where *p* < 0.05 was considered significant.

## Results

### [^68^Ga]Ga-DOTA-TATE uptake in pilot LPS model

**Fig. 2:**
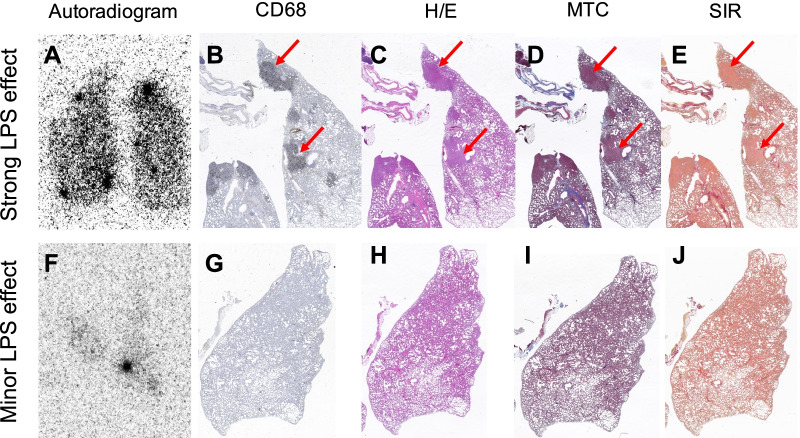
Ex vivo autoradiography uptake of [^68^Ga]Ga-DOTA-TATE and in lungs from rat with strong lung injury (**A**) and infiltration of CD68 + macrophages (**B**) as verified by biopsy histology (C-E), as well as a rat with minor lung injury free of macrophages (**F–J**). The abbreviations for the stainings are as follows: Hematoxylin/ Eosin (H/E), Massons Trichrome (MTC), Sirius Red (SIR)

In a pilot experiment, rats were treated with LPS to induce lung inflammation. For the MRI and CT data, see Additional file 1: Fig. S1. Based on the CT and MRI scanning of the LPS treated rats in the pilot study, radiological signs of lung inflammation were visible after 24 h, and this time point was selected for further PET imaging and ex vivo organ distribution studies. After administration of [^68^Ga]Ga-DOTA-TATE, the in vivo signal in lung was studied by ex vivo autoradiography (Fig. 2). The efficacy of the LPS induction varied between the animals, e.g., due to variable exposure of the toxin to the tissue, as assessed by histology of the lung (Fig. 2). Interestingly, the lung uptake as assessed by ex vivo autoradiography also varied between the animals, and in the animal with pronounced lung inflammation and infiltration of CD68 positive macrophages (Fig. 2A–E), a similar strong and heterogeneous binding of [^68^Ga]Ga-DOTA-TATE was observed. In a rat with minor LPS effect and negligible lung injury, correspondingly low binding of [^68^Ga]Ga-DOTA-TATE was seen (Fig. 2F–J). Representative images of the [^68^Ga]Ga-DOTA-TATE uptake on sagittal (Additional file 1: Fig. S2A) and coronal (Additional file 1: Fig. S2B) view display that most uptake is located at the main bronchi area, end of trachea and area of thymus.

### [^68^Ga]Ga-DOTA-TATE uptake in rat LPS model

**Fig. 3:**
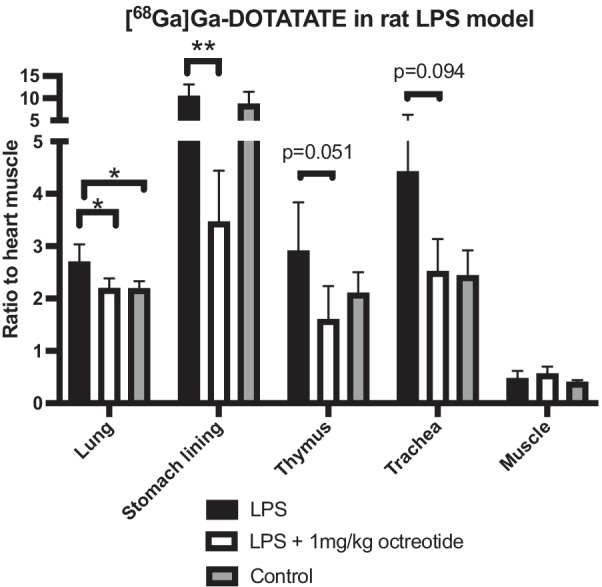
Ex vivo biodistribution of lung, stomach, thymus, trachea and muscle [^68^Ga]Ga-DOTA-TATE heart muscle ratio uptake on rats. First group was treated with LPS (black) and shows significant difference in lungs and stomach compared with second group treated with LPS together with blocking with octreotide (white). The LPS-treated group also showed a significant difference compared with control group (grey) in the lungs, but not in stomach, thymus or trachea. The negative control tissue muscle had significantly lower uptake in the LPS group compared with lungs (*p* < 0.0001), thymus (*p* < 0.001) and trachea (*p* < 0.01)

Based on the promising results in the pilot rat LPS model, we evaluated [^68^Ga]Ga-DOTA-TATE in a larger study. The biodistribution of [^68^Ga]Ga-DOTA-TATE was assayed in lung injury model where endotoxin LPS causes pro-inflammatory response of macrophages (Fig. 3). The accumulation of [^68^Ga]Ga-DOTA-TATE in the lungs of LPS treated animals was increased compared to untreated control animals without lung injury. (Fig. 3 and Additional file 1: Fig. S3). Importantly, the increased binding in LPS-treated rats could be attenuated by the pre-treatment with octreotide, indicating a SSTR2 mediated binding of [^68^Ga]Ga-DOTA-TATE. Binding in SSTR2 positive tissues such as stomach lining and thymus was high and inhibited by octreotide in both the LPS and the non-treated control rats. Binding in lung, stomach, thymus, and trachea was increased after 24 h of treatment of LPS compared with negative control tissue muscle. Apparent increase in thymus and trachea uptake did not reach statistical significance between the LPS and blocking (thymus *p* = 0.051, trachea *p* = 0.0945) or control groups.

### [^68^Ga]Ga-DOTA-TATE uptake in pig lavage model

**Fig. 4:**
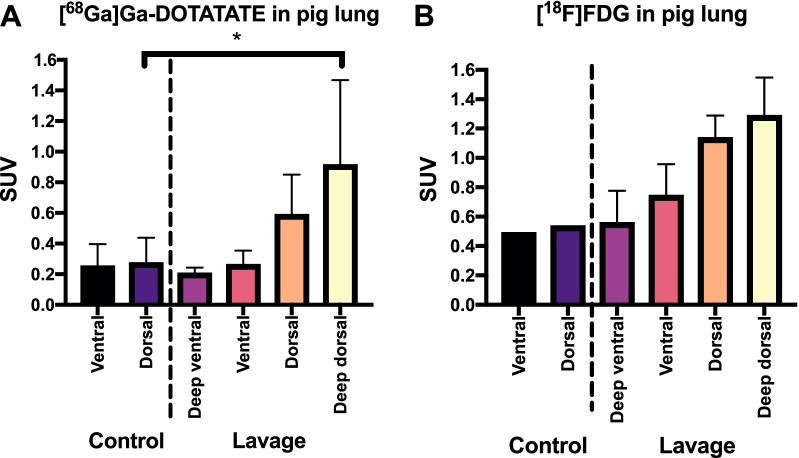
Comparison of the PET uptake of (**A**) [^68^Ga]Ga-DOTA-TATE and (**B**) [^18^F]FDG in healthy control and pigs with lung inflammation at 60 min p.i. The [^68^Ga]Ga-DOTA-TATE uptake was significantly increased on the deep dorsal parts of the lungs on the pigs induced with lavage, compared with the control animals. The uptake of [^18^F]FDG is elevated on the control and sick animals in comparison with the [^68^Ga]Ga-DOTA-TATE uptake. With both tracers, the SUV increased toward the deep dorsal parts of the animals with ARDS. The uptake on control animals remained on the same level on ventral and dorsal segments

After demonstrating the uptake of [^68^Ga]Ga-DOTA-TATE in lung inflammation on rats, a large animal model was used. A lavage induced lung damage was assessed on pigs, in comparison with healthy animals. Severe lung inflammation was verified by continuing induction until p/f ratio was below 100 (classified as severe ARDS). In order to see larger scale of the inflammation in the lungs, the uptake of [^18^F]FDG was also monitored in some of the animals. The segmentations of the lungs (Fig. 4) were divided into four parts on the sick pigs according to the anatomy: deep ventral lavage, ventral lavage, dorsal lavage and deep dorsal lavage and in two parts on the control animals: ventral and dorsal. Similarly, biopsies were taken from the same lung regions for histology (Additional file 1: Fig. S4). The more damaged deep dorsal parts (SUV_mean_ = 0.91 ± 0.56, *p* < 0.05, *p* = 0.025) demonstrated significantly higher uptake of [^68^Ga]Ga-DOTA-TATE (Fig. 4A) compared with the control pigs (SUV_mean_ = 0.27 ± 0.16) at 60 min p.i. The [^18^F]FDG uptake (Fig. 4B) was expectedly higher in both healthy and ARDS animals compared with [^68^Ga]Ga-DOTA-TATE.

**Fig. 5:**
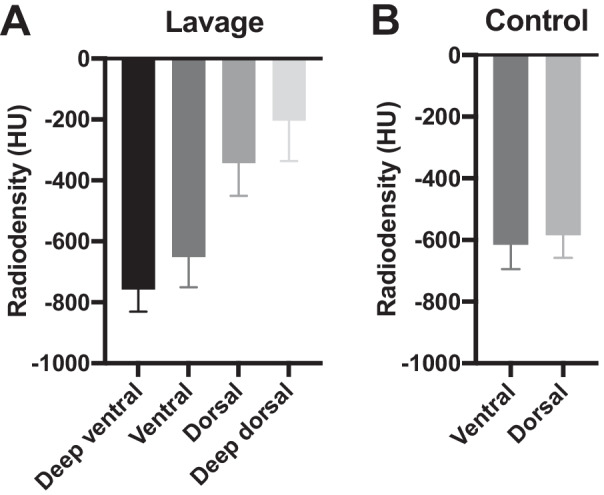
CT HU values of lavage induced and control pigs. The HU values increased toward the deep dorsal parts on the sick pigs and remained on the same level for the control animals on both ventral and dorsal segments

Using the same segregation of the volumes of interest (VOI’s) on CT images, the HU were also compared on sick lavage and control pigs (Fig. 5). In line with the SUV from PET images, the HU showed increase toward the dorsal parts of the animal, indicating higher density. For the healthy animals, the HU remained around -600 on both ventral and dorsal parts.

**Fig. 6:**
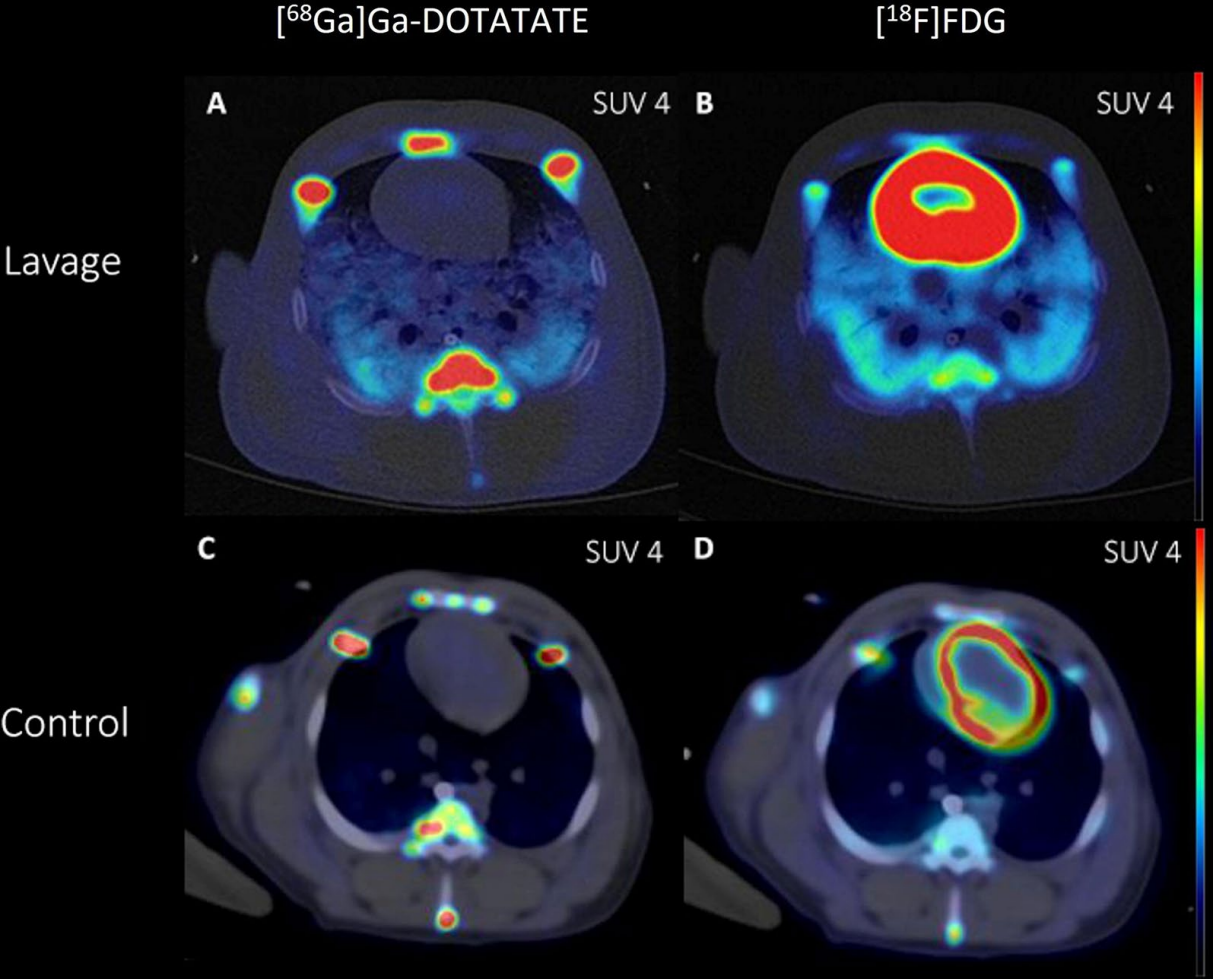
Representative fused PET-CT images of (**A,** **C**) [^68^Ga]Ga-DOTA-TATE and (**B,** **D**) [^18^F]FDG uptake in lavage (**A,** **B**) and control (**C**, **D**) pigs. Signal quantification is presented as summary of 30 min-60 min p.i. in SUV_mean_ scale

In agreement with the results of quantification, the PET-CT images (Fig. 6) displayed increased uptake in the damaged lungs (Fig. 6A, B), while the uptake in healthy lungs of the pigs (Fig. 6C, D) was neglectable. The damage was also visible on the fused CT images. The elevated accumulation of [^18^F]FDG (Fig. 6B, D) in the heart is typical due to the high glucose metabolism. In the bone, increased activity was evident on both tracers and on sick and healthy animals.

## Discussion

Acute inflammation plays a central role in several pulmonary disorders including acute respiratory distress syndrome (ARDS) [20, 21]. The current evaluation of lung damage mainly depends on functional parameters or histological measurements, which do not offer specific regional information and cannot be used for longitudinal monitoring of the disease. The grade of inflammatory response together with cellular changes during the early stages of pulmonary inflammation is indicated to reflect the prognosis [22]. Hence, early identification of the tissue-specific cell types and longitudinal follow-up could help physicians to monitor patients’ response to therapy and increase the chances of survival [23].

In this study, our aim was to further investigate the potential and mode of action of [^68^Ga]Ga-DOTA-TATE to detect activated macrophages during acute lung inflammation. Several clinical studies have demonstrated the potential of using [^68^Ga]Ga-DOTA-TATE for PET imaging of inflammatory foci, especially in the setting of atherosclerosis [8–10]. Furthermore, recent data by Tarkin et al. demonstrated that SSTR2 was upregulated specifically on macrophages with a M1 polarized phenotype [8], while absent from other macrophages. Additionally, other SSTR subtypes were not upregulated on the M1 polarized macrophages. Thus, it is reasonable to assume that [^68^Ga]Ga-DOTA-TATE accumulation in regions with active inflammation is mediated primarily by binding to the SSTR2 subtype. However, this mechanism has merely been implied and not demonstrated, as blocking studies with SSTR2 binders are challenging in the clinical setting (and ex vivo studies are impossible). This study therefore set out to investigate the performance of [^68^Ga]Ga-DOTA-TATE in several animal models of inflammation and macrophage recruitment, where blocking studies and post-mortem staining is feasible as complement to PET imaging.

To our knowledge, [^68^Ga]Ga-DOTA-TATE has not been used to study uptake in animal models of pulmonary dysfunction before. We tested the uptake in rat LPS model together with blocking and pig ARDS lavage model. The LPS model was selected as it is widely established, and directly activates the macrophages that upregulates SSTR2. In human macrophages, the SSTR2 upregulation mainly mediates immunosuppressive effect of somatostatin, and in tumors, it inhibits the growth by affecting the cellular level signaling molecules in apoptotic pathway, MAPK pathway and angiogenesis.

From translational point of view, the main differences of rat and pig lungs are the horizontal airways compared with the mostly vertical airway in humans and the respiratory frequency, which is higher on small animals. The anatomy of the bronchial tree on human is dichotomous and on pig and rat monopodial, which explains the higher efficiency of the air flow on rats and pigs. Rats have 4 times higher oxygen uptake than man [24].

The ratio to heart muscle of [^68^Ga]Ga-DOTA-TATE in the LPS treated rat lungs was significantly higher when compared with blocking and control groups, indicating specific uptake. The heart muscle was used as reference tissue to normalize against, as myocardium is devoid of SSTR2. The other immune organ thymus and the route of LPS induction through trachea also had clear tendency for increased uptake, though they did not significantly differ from the control group. The variability in tracer binding seen between individual animals is likely primarily due to the different exposure to LPS in the lung. In our experience, there is some unavoidable variation in the regional lung exposure (using radioactivity and dyes for detection) even for well-trained personnel performing oropharyngeal administration. The LPS models on mice and rat have otherwise been frequently used in research and is considered one of the most established animal models for lung inflammation [25]. Interestingly, there is a great variability on the sensitivity to LPS between humans and rats. To induce a severe disease leading into shock, we humans can handle nearly 7000 times less dose than a rat [26]. However, the in vitro studies with similar concentrations of LPS show similar sensitivity in activation of cytokines on both species. According to Warren et al., this is due to mouse serum’s ability to inhibit cytokine signaling (TNF and IL-6) instead of differences in cell populations between species. While these findings considered to a lesser extent alveolar macrophages, it suggests that macrophages might behave differently in vitro than in vivo. Despite of the results in this study, the in vivo activation of pro-inflammatory macrophages by LPS may not be as intense on rats and mice as previously supposed, and other small animal models should be considered for the future studies of macrophage targeting inflammation tracers [27].

Due to the possible translational discrepancies between humans and rat LPS model, we moved on to a large animal model. The porcine immune system and alveolar macrophages present more similar behavior to human than rat or mice [28]. However, since that subclinical respiratory infections are relatively common in farm pigs, the healthy control animals could also have underlying conditions and should thus be considered with some caution [29]. We imaged farm pigs with lung lavage model using [^68^Ga]Ga-DOTA-TATE and [^18^F]FDG and quantified the SUV uptake and HU of CT scans. Since most inflammatory cells have increased glucose uptake, [^18^F]FDG has already been established as a useful tool to identify inflammatory processes. Increased glucose metabolism is also part of pro-inflammatory macrophage response; however, the tissue resident alveolar macrophages do not promote increased glycolysis as intensively as the bone-marrow recruited ones do [30]. This could be due to the natural environment of tissue resident macrophages in alveolar lumen, where the glucose concentration is constantly low; less than 10% of the blood glucose concentration [31]. This limits the use of [^18^F]FDG for identification of macrophage targeting therapies [32, 33].

Among other emerging techniques for imaging of macrophages, it is worth to specifically mention PET radioligands targeting the folate receptor [34, 35]. This approach has been extensively evaluated in preclinical models, e.g., of arthritis and myocarditis, and recently a first-in-man trial in rheumatoid arthritis was reported [35]. To the authors knowledge, folate receptor PET has not yet been evaluated in models of pulmonary inflammation. We believe folate receptor PET may potentially be more macrophage specific than [^68^Ga]Ga-DOTA-TATE, but the wide availability of clinical grade [^68^Ga]Ga-DOTA-TATE at PET centers worldwide offers unique benefits by potentially enabling straightforward clinical deployment at many sites. A direct comparison between these approaches is clearly warranted.

In our study, the uptake of [^68^Ga]Ga-DOTA-TATE in lavage pigs was significantly increased in the more damage parts of the lungs compared with healthy control animals. The HU quantification and [^18^F]FDG uptake were in line with the results. We also noticed increased uptake in the bone of the pigs with induced lung inflammation with both tracers, which was more visually intense on [^68^Ga]Ga-DOTA-TATE images. Initially, we considered that this may be reactive uptake in response to increased bone marrow hematopoietic function. On the other hand, a similar high uptake of [^68^Ga]Ga-DOTA-TATE was also seen in bone of control pigs, without induced inflammation. Furthermore, similar strong binding was not seen in bone in rats. Thus, a more likely reason is that the strong bone accumulation of [^68^Ga]Ga-DOTA-TATE is specific for the young pigs used in this study. This could perhaps reflect high macrophage activity in growth plates and could open up the future use of [^68^Ga]Ga-DOTA-TATE, e.g., in osteoarthritis and biomaterial studies.

## Conclusions

Our data substantiates that [^68^Ga]Ga-DOTA-TATE is capable to assay tissue macrophages noninvasively in both porcine and rat lung models. This mechanistic understanding, combined with the increased data gathered from several inflammation studies on humans, indicates that [^68^Ga]Ga-DOTA-TATE has potential to be used to study the role and presence of macrophages in humans in the fight against severe lung diseases.

## Supplementary Information

**Additional file 1.** Contains detailed information of the radiolabeling procedure as well as supplementary figures.

## Data Availability

The datasets used and analyzed during the current study are available from the corresponding author on reasonable request.
