# Supplementary material for: PET-CT imaging of pulmonary inflammation using [68Ga]Ga-DOTA-TATE
Source: EJNMMI Res. 2022 Apr 8;12:19. doi: 10.1186/s13550-022-00892-0 (PMC8994000; doi:10.1186/s13550-022-00892-0)
Supplement: Supplementary file 1 — Additional file 1. Contains detailed information of the radiolabeling procedure as well as supplementary figures. [file 13550_2022_892_MOESM1_ESM.docx]

**Additional file 1**

**Supplementary data, PET-CT imaging of pulmonary inflammation using [^68^Ga]Ga-DOTA-TATE**

# MATERIALS AND METHODS

## *Radiosynthesis of [^68^Ga]Ga-DOTA-TATE*

Top fraction of 68Ge/68Ga generator (50 mCi, Eckert and Ziegler, Eurotope GmbH) eluate was collected containing 80–85% (range 700-850 MBq) of the total 68Ga radioactivity in 3.0–3.5 mL of 0.1 M hydrochloric acid. The eluate was buffered with a 250–350 µL of sodium acetate buffer (1M, pH 4.6) and 25-30 µL of sodium hydroxide (10M) providing a pH of 4.6–5.0 prior to the addition of 15-20 nanomoles (1 mM) of DOTA-TATE (ABX, Germany). The mixture was incubated in a 10 mL glass vial in a heating block at 75 °C for 10-15 min. The water was added to the crude product solution for cooling and subsequent purification on solid-phase extraction using disposable cartridges (C8, Waters). The product was recovered with 1 mL of 50% ethanol solution and formulated in phosphate buffer to assure the content of ethanol less than 10%. A high-performance liquid chromatography system (HPLC, Agilent Technologies 1200 system) consisting of a 1290 pump, 1290 Vialsampler,1260 Variable Wavelength Detector (UV), and a radiation flow detector (Bioscan) coupled in series was used for the quality control of the product and determination of the peptide concentration. A reversed phase analytical column (ACE, C-18, Scantec Nordic) was used for the separation of the analytes under the following conditions: A = 10 mM TFA in water; B = 100% acetonitrile/10mM TFA with UV-detection at 220 nm; linear gradient elution: 0–8 min from 24 to 60% B, 8–14 min 60% B; flow rate was 0.6 mL/min. Data acquisition and handling were performed using the OpenLAB Software Package. The radioactivity recovery from the HPLC column was confirmed by conducting analysis with and without column, collecting the mobile phase from the outlet and measuring its radioactivity. The readings from an in-house built well-type NaI(Tl) scintillation counter used for the radioactivity measurement was corrected for dead-time and decay. The retention time for the radio-signal of [68Ga]Ga-DOTA-TATE was 3.8 ± 0.2 min.

**FIGURES**

**Supplementary figure S1.** MRI and CT images of the development of LPS induced lung inflammation in rat. The rats were imaged at baseline before induction of LPS and at 24h, 48h and 72h post-administration. The upper row (A) represents the longitudinal MRI images and lower row (B) CT images of the same rat. The density on lungs (yellow arrow) increases visually mostly one sided after 24h post-administration and remains roughly the same at 48h and 72h.


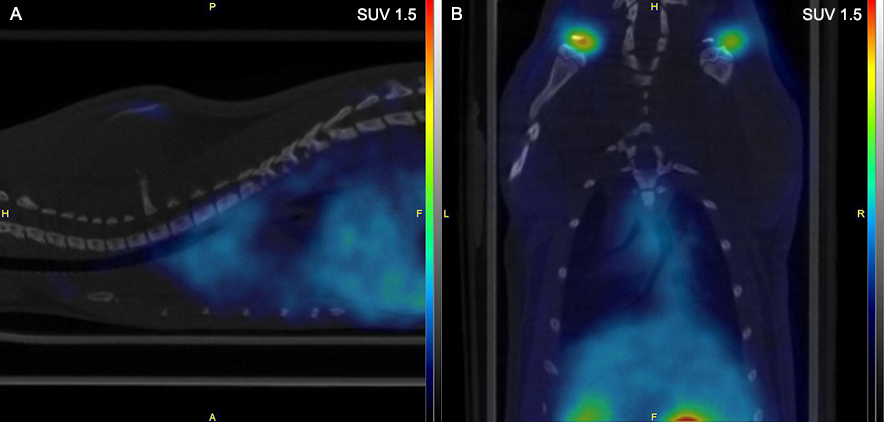


**Supplementary figure S2.** Representative (A) sagittal and (B) coronal images of LPS induced rat at 10-30min p.i. The uptake is mainly focused around the main bronchial area and at the end of trachea.

**Supplementary Figure S3.** Representative [^68^Ga]Ga-DOTA-TATE binding to lung and thymus of untreated control rats (A), LPS treated rats (B) and after octreotide pretreatment in LPS treated rats (C). Representative staining for CD68, Hematoxylin/ Eosin (H/E) and Sirius Red (SIR) in each group are shown.

**Supplementary Figure S4.** Representative staining of lung PFA biopsies from pig with ARDS (top panels) and healthy untreated control (bottom panels). The abbreviations for the stainings are as follows: Hematoxylin/ Eosin (H/E), Massons Trichrome (MTC), Safranin O (SaffrO), Sirius Red (SIR), Alcian Blue-van Giessen (AB-vG) and Alcian Blue Periodic Acid-Schiff (AB-PAS)
